# On the perils of working on nonmodel organisms

**DOI:** 10.1073/pnas.2216683120

**Published:** 2023-03-09

**Authors:** Maria Pia Miglietta

**Affiliations:** ^a^Department of Marine Biology, Texas A&M University at Galveston, Galveston, TX 77553

In Vol 119, No. 36 of PNAS, Pascual-Torner *et al*. (1) compare the genome of the “immortal jellyfish” *Turritopsis dohrnii* with that of *Turritopsis rubra* under the rationale that because *T. rubra* is “incapable of postreproductive regeneration”, the comparison will uncover the unique genomic characteristics that allow *T. dohrnii's* rejuvenation. The framework of the paper is based on unfounded assumptions, which calls the paper's conclusions into question.

The assertion that *T. rubra* is incapable of postreproductive regeneration is incorrect. Pascual-Torner et al. support it by referencing a paper (2) that never tested the ability of *T. rubra* to regenerate before or after reproduction, as it focuses on a different species, the Chinese *T.* sp. 5 (2). The articles that have evaluated the rejuvenation of *T. rubra* (3–5) are not cited by Pascual-Torner. These articles focus on a Japanese *Turritopsis*, presumably *T. rubra*, and conclude that *T. rubra* can rejuvenate both before and after reproduction, though at a lower rate. How does this impact the study's findings? As the premise of the comparative analysis is incorrect, so is the claim that comparing the species' genomes can “unveil crucial molecular pathways driving rejuvenation.” The fact that both species can rejuvenate before and after reproduction implies that both possess the genetic machinery to do so. Comparing their genomes cannot identify the keys to rejuvenation. It is similarly problematic to suggest, as the authors do, that their results should inform future research in mammalian systems.

The second part of the paper focuses on the transcriptome of the life-cycle stages of *T. dohrnii*. Although depicted as novel, there are currently two published transcriptomes of *T. dohrnii's* life cycle stages (6, 7) that are not discussed, a critical omission as both studies provide relevant results. Moreover, in Pascual-Torner et al., reversal was induced by inconsistent methods (CsCl and “spontaneously”), which may result in differences in gene expression patterns. Finally, what the authors call the “no-reversal stage” is most likely a medusa that is taking longer to revert to a polyp (common and dependent on medusa size) and not a medusa that is failing to revert (impossible to determine *a priori*, as the only way to know is for the medusa to die and decompose).

Finally, when working with understudied non-model organisms, correct species identification is crucial. With cryptic species and species introductions, misidentifications are frequent in the genus *Turritopsis* (2, 8). The burden of showing that the species whose genomes are sequenced are correctly identified falls on the authors. This paper gives no information on the author(s) of the identification, how these species were identified, or the availability of voucher specimens.

As genomic and transcriptomic research moves beyond a handful of traditional, well-known model organisms, it is of paramount importance that both authors and reviewers are aware of the dangers of tackling the genomes of nonmodel organisms without a clear understanding of their biology, that taxonomists are adequately consulted to ensure correct species identification, and that all pertinent literature is correctly interpreted and referenced.
